# Diversity and Seasonal Dynamics of Stored-Product Insects in a Feed Manufacturing Facility in Greece

**DOI:** 10.3390/insects16121209

**Published:** 2025-11-27

**Authors:** Evagelia Lampiri, Paraskevi Agrafioti, Efstathios Kaloudis, Dimitrios Kateris, Christos G. Athanassiou

**Affiliations:** 1 Laboratory of Entomology and Agricultural Zoology, Department of Agriculture, Crop Production and Rural Environment, University of Thessaly, Phytokou Str., 38446 Volos, Greece; agrafiot@uth.gr (P.A.); athanassiou@uth.gr (C.G.A.); 2 Institute for Bio-Economy and Agri-Technology (IBO), Centre for Research and Technology—Hellas (CERTH), Dimarchou Georgiadou 118, 38333 Volos, Greece; d.kateris@certh.gr; 3 Computer Simulation, Genomics and Data Analysis Laboratory, Department of Food Science and Nutrition, School of the Environment, University of the Aegean, Ierou Iochou 10 & Makrygianni, 81400 Myrina, Greece; stathiskaloudis@aegean.gr

**Keywords:** stored-product insects, animal feed industry, insect monitoring, dominance and frequency, seasonal population dynamics

## Abstract

The present study aimed to document the diversity and seasonal dynamics of stored-product insects in an animal feed facility located in northern Greece. A total of 38 traps were installed across different operational areas of the facility and inspected over 51 consecutive sampling occasions. Captured insects were identified to the lowest possible taxonomic level, and their frequency and dominance were calculated. In total, 9047 insect species belonging to five orders, 14 families, and at least 18 insect species were recorded. The dominant species were *Tribolium castaneum*, *T. confusum*, *Oryzaephilus surinamensis*, *Sitophilus granarius*, *Lasioderma serricorne*, and Lepidoptera adults, which collectively accounted for more than 85% of all captures. The total number of insects exhibited marked seasonal fluctuations, with the highest captures during late summer and early autumn and minimal activity during winter. Positive and significant correlations were detected among several dominant species, notably between Lepidoptera and *T. castaneum*, suggesting overlapping environmental preferences within the facility. These findings provide a detailed overview of the insects associated with feed industries in Greece and underscore the importance of continuous monitoring for effective pest management. The results highlight the need for seasonally adjusted control measures and contribute to a better understanding of the dynamics of stored-product pests under industrial conditions.

## Simple Summary

This study investigated the insects found in an animal feed facility in northern Greece over a two-year period. Using 38 traps placed throughout the facility, insects were collected and identified during 51 inspections. In total, 9047 insects were recorded. Only a few species were found to be dominant and in high numbers, mainly *Tribolium castaneum*, *T. confusum*, *Oryzaephilus surinamensis*, *Sitophilus granarius*, *Lasioderma serricorne*, and adult Lepidoptera. The number of insects changed throughout the year, with the highest captures in late summer and early autumn and very low numbers during winter. Some species were often found together, showing that they prefer similar conditions inside the facility. The study provides useful information about which insects are most common in feed industries and when they are most active, helping to improve pest monitoring and control strategies.

## 1. Introduction

Stored-product insects represent one of the most persistent and economically significant threats to the preservation of agricultural commodities and processed goods worldwide. These species infest a wide range of products, including grains, flour, dried fruits, and animal feed, and are responsible for both quantitative and qualitative losses [1,2]. Infestations reduce the nutritional value of commodities, contaminate them with frass and insect fragments, and often facilitate the growth of secondary pathogens such as fungi [3,4,5,6]. Beyond direct product losses, the presence of insects in storage facilities and feed industries raises serious concerns related to food safety regulations and consumer acceptance [7].

Monitoring insect populations in stored-product environments is a cornerstone of integrated pest management (IPM) [8,9,10]. Trapping is the most widely used method for detecting and quantifying insect presence, providing valuable insights into species composition, population fluctuations, and spatial distribution within storage facilities [11,12]. Long-term monitoring enables researchers and practitioners to identify seasonal patterns, assess the impact of environmental factors such as temperature and humidity, and implement targeted control strategies. In industrial facilities, such as feed mills, where raw materials of diverse origin are stored and processed, trapping becomes even more critical, as the risk of cross-contamination and reinfestation is substantial [13].

Previous studies in Europe and worldwide have documented the diversity of insect communities in storage ecosystems. For instance, *Sitophilus* spp. (Coleoptera: Curculionidae), *Tribolium* spp. (Coleoptera: Tenebrionidae), *Oryzaephilus* spp. (Coleoptera: Silvanidae), the lesser grain borer, *Rhyzopertha dominica* (F.) (Coleoptera: Bostrychidae), and the cigarette beetle, *Lasioderma serricorne* (F.) (Coleoptera: Anobiidae), are commonly reported as primary and secondary pests of grains and processed products [14,15]. Surveys in silo structures have demonstrated that insect distribution is often heterogeneous, with localized foci of infestation driven by microclimatic conditions and product type [15]. Similar findings have been reported in another feed industry setting study, where monitoring revealed a mixture of cosmopolitan species with varying degrees of dominance and frequency [16]. In Greece, research on the entomological load of stored products has traditionally focused on grain silos, warehouses, and processing facilities, highlighting the presence of key cosmopolitan species, as well as the influence of climatic and operational conditions on population dynamics [16,17].

Despite the available knowledge, there is still limited information on the insect populations associated with the animal feed industry, where large quantities of raw materials are processed, mixed, and stored under conditions that may differ substantially from conventional grain storage. Feed mills represent complex environments characterized by high turnover of materials, frequent movement of goods, and multiple entry points for pests. Consequently, the dynamics of insect populations in such facilities may not necessarily reflect those observed in silo or warehouse conditions [18]. Moreover, the role of spatial and temporal factors in shaping the occurrence of insect species within feed industries has not been adequately documented.

Given these considerations, the present study aimed to record the insect populations in a feed industry in Greece, focusing on the spatial and temporal distribution of captures in traps over an extended monitoring period. By documenting species presence, frequency, and dominance patterns, the study aims to contribute to a better understanding of the entomological risks faced by feed industries. The findings are expected to provide useful insights into the development of improved monitoring protocols and integrated pest management strategies tailored to industrial storage and processing environments.

## 2. Materials and Methods

### 2.1. Facility Location

The study was conducted in a feed industry located in Northern Greece. The facility consisted of multiple storage and processing areas, including raw material storage, production lines, and finished product warehouses. The particular feed industry processed and stored both hard and soft wheat, as well as smaller amounts of barley and maize. The study site was selected due to its continuous operation and the presence of diverse raw materials of plant origin, which provide suitable substrates for insect infestation. Environmental conditions were not artificially regulated and followed seasonal variations.

### 2.2. Trapping Design and Monitoring

**Figure 1:**
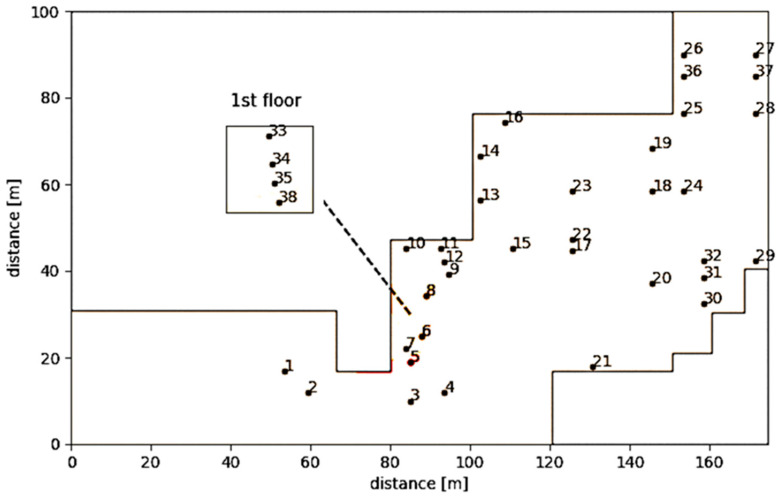
The location and the label number of the traps set up in the feed industry.

On 15 July 2021, the traps were installed within the facility (Figure 1), positioned on both the first and basement floors. A total of 38 traps were deployed throughout the facility to monitor insect activity. Traps were placed in locations representing different functional areas, including production halls, packaging, and distribution areas. The traps included 30 StorgardTM oil kairomone food (Oil, Trécé Inc., Adair, OK, USA) -baited dome traps (Trécé Inc., Adair, OK, USA) and 8 pheromone-baited delta traps (StorgardTM, Trécé Inc., Adair, OK, USA), depending on the target insect groups. Trap numbers 2, 7, 9, 22, 23, 36, 37, and 38 were delta traps, while all the others were dome traps. The delta traps’ placement was standardized at a height of 180 cm from the ground and positioned away from direct airflow or obstacles to ensure comparability.

Monitoring was carried out over a period of 2 years, from July 2021 to June 2023, with 51 consecutive sampling dates at biweekly intervals, except times when periodic pesticide spraying in the industry facilities using Actellic 50 EC (48% AI, Syngenta Hellas, Athens, Greece), which was acquired from Syngenta International AG (Basel, Switzerland), restricted access to trapping areas. In this study, each sample refers to the capture from an individual trap during a single inspection date. Weather data were obtained from the Athens National Observatory (https://meteosearch.meteo.gr, accessed on 17 November 2025). During each inspection, all traps were examined, captured insects were collected, and traps were refreshed or replaced as needed. The number of insects per species was recorded, and specimens were transported to the Laboratory of Entomology and Agricultural Zoology (LEAZ), Department of Agriculture, Crop Production and Rural Environment, at the University of Thessaly, for further identification according to distinct taxonomic keys.

### 2.3. Insect Identification

Captured insects were identified to the lowest possible taxonomic level (species or genus) using standard morphological keys [19,20,21]. Adults of major stored-product pests, such as the confused flour beetle, *Tribolium confusum* Jaquelin duVal (Coleoptera: Tenebrionidae), the red flour beetle, *Tribolium castaneum* Herbst (Coleoptera: Tenebrionidae), the granary weevil, *Sitophilus granarius* (L.) (Coleoptera: Curculionidae), the sawtoothed beetle, *Oryzaephilus surinamensis* (L.) (Coleoptera: Silvenidae), and *R. dominica*, were identified to the species level. Other taxa (e.g., Carabidae, Diptera, Hymenoptera) were identified at family or order level when morphological characters did not allow precise identification. Lepidoptera adults captured in the delta traps were attracted by a pheromone lure designed to target both the Indianmeal moth, *Plodia interpunctella* (Hübner) (Lepidoptera: Pyralidae) and *Ephestia* spp. (Lepidoptera: Pyralidae). In addition, a proportion of the captured individuals were damaged inside the traps, preventing the use of morphological characters required for reliable species-level identification. Therefore, all *P. interpunctella* and *Ephestia* spp. adults were recorded collectively as Lepidoptera.

### 2.4. Data Analysis

Curry’s [22] and Buchelos and Athanassiou’s [23] “Dominance” and “Frequency” criteria were used in the analysis of the collected insect populations. The percentage of insects in a given species relative to the total number of insects in all species is known as dominance. Based on this, a species can be categorized as Dominant, Influent, or Recedent, which correspond to >5, 2–5, and <2% of all insects recorded, respectively. A species’ “frequency” is determined by the proportion of samples that contain reports of that species. Accordingly, a species can be categorized as Accidental, Accessory, or Constant if insects of that species account for less than 25%, 25–50, or more than 50% of all samples, respectively [23]. Additionally, correlation coefficient values were computed to assess the “synchronization” between pairs of captures among the dominant insect species on the same date. A two-tailed t-test was used to determine if these values deviated from zero at the n–2 *df* and 0.01 significance level. Furthermore, the Matplotlib package (version 3.10.3) for visualization and the SciPy library’s (version 1.15.3) interpolation module for processing data points in many dimensions were used to depict the spatiotemporal distribution using Python (version 3.12).

## 3. Results

A total of 9047 adult insects were captured during the monitoring period across all 38 traps. Five orders belonging to 14 families and at least 18 species were identified (Table 1). Among them, six species were classified as dominant based on their relative abundance: *T. castaneum*, *T. confusum*, *O. surinamensis*, *S. granarius*, *L. serricorne*, and Lepidoptera adults. All other taxa were recorded as influent or recedent species. The most abundant group was Lepidoptera (adults), which accounted for 45.11% of all insects collected. *Tribolium castaneum* followed with 12.91%, while *L. serricorne* and *O. surinamensis* represented 10.95% and 6.33%, respectively. *T. confusum* and *S. granarius* comprised 5.32% and 7.42% of the total captures, respectively (Table 1). Species or orders such as the drugstore beetle, *Stegobium paniceum* (L.) (Coleoptera: Anobiidae), the rice weevil, *Sitophilus oryzae* (L.) (Coleoptera: Curculionidae), and Hymenoptera were classified as influent with low dominance values (2–5%). The remaining taxa were found sporadically and represented less than 1% of total captures, categorized as recedent (Table 1).

In terms of frequency, Lepidoptera adults were the most frequently detected taxon, occurring in 19.09% of all samples, followed by *T. castaneum* (15.12%), *O. surinamensis* (11.04%), *S. granarius* (9.34%), and *T. confusum* (9.13%). All dominant species were classified as accidental, reflecting moderate to low consistency across samples (Table 1).

**Figure 2:**
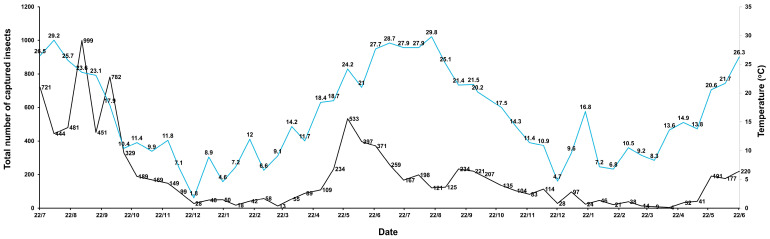
Total number of captured insects and temperature across the monitoring period. The black line indicates the number of captured insects, while the blue line indicates the temperature.

The total number of captured insects varied substantially throughout the monitoring period, according to temperature fluctuations (Figure 2). Captures were the highest during the first months of the study, with a clear peak in early September 2021 (999 insects). After this initial maximum, numbers progressively declined toward late autumn and winter, as the temperature declined, reaching their lowest levels between March and April 2023 (below 10 insects). A secondary increase was recorded during the final sampling months, with captures reaching 220 insects in June 2023. Overall, total captures followed a seasonal and temperature pattern, with elevated activity during the warmest months and low abundance during the coldest period (Figure 2).

**Figure 3:**
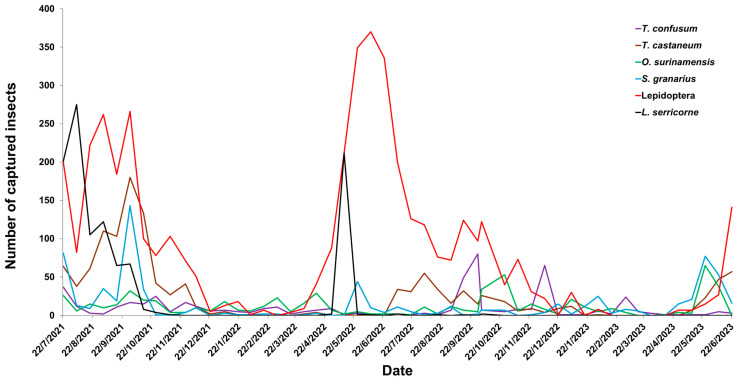
Population fluctuation in the dominant insect species, *Tribolium confusum*, *Tribolium castaneum*, *Oryzaephilus surinamensis*, *Sitophilus granarius*, Lepidoptera, and *Lasioderma serricorne* during the monitoring period.

The population dynamics of the dominant species followed similar but distinct temporal trends (Figure 3). Lepidoptera adults consistently represented the most abundant group, showing several pronounced peaks, particularly in September 2021 and again in early 2022. *L. serricorne* and *T. castaneum* exhibited moderate but recurrent activity peaks during late summer and autumn. At the same time, *O. surinamensis* and *S. granarius* showed more irregular fluctuations, with increased captures in early and late 2022. *Tribolium confusum* was generally less abundant, with sporadic captures and no distinct seasonal trend (Figure 3).

The correlation analysis among the dominant insect species revealed generally weak to moderate relationships in trap captures (Table 2). *Tribolium confusum* showed minimal correlations with all other species, indicating largely independent occurrence patterns. In contrast, *T. castaneum* exhibited significant positive correlations with most species, including *O. surinamensis*, *S. granarius*, and Lepidoptera, except for *L. serricorne*, for which the correlation was not significant (R = 0.33, *t =* 0.462, *P =* 0.64). Notably, all combinations involving Lepidoptera were significant and positive. Other species pairs displayed weak or non-significant correlations, indicating largely independent capture patterns (Table 2).

**Figure 4:**
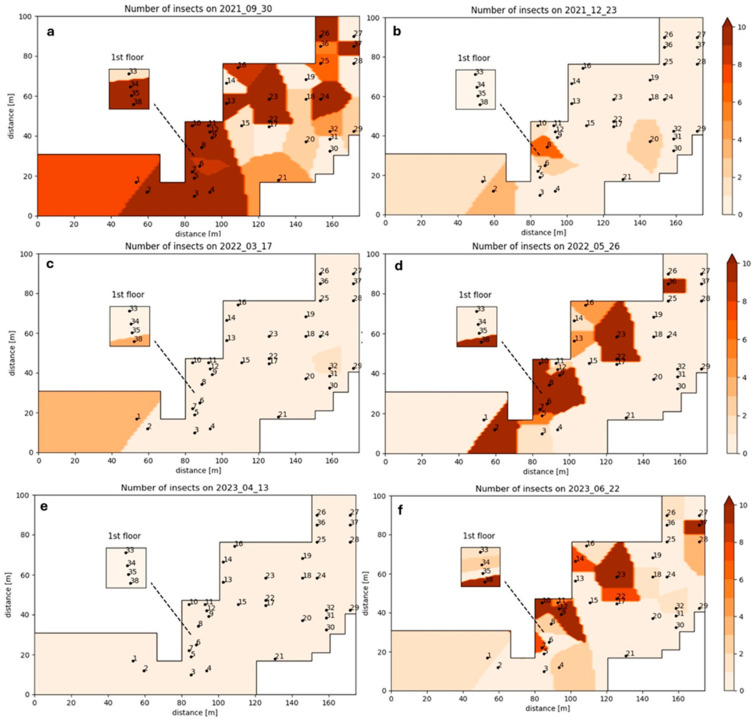
Spatial distribution of the insects inside the feed industry on (**a**) 30 September 2021, (**b**) 23 December 2021, (**c**) 17 March 2022, (**d**) 26 May 2022, (**e**) 13 April 2023, and (**f**) 22 June 2023.

The spatial distribution of insect captures showed clear temporal and spatial variation across the facility. During the first sampling (Figure 4a), insect abundance was high and widespread, with the highest densities concentrated in the central zone corresponding to the production and processing line and extending toward the northeastern storage areas. In December 2021 (Figure 4b), captures remained relatively high but were more localized around the processing line, indicating a contraction of the infestation focus during the colder period. In March 2022 (Figure 4c), insect captures were minimal across both production and storage areas, reflecting a seasonal decline in insect activity during winter. By May 2022 (Figure 4d), insect densities increased again, primarily in the central processing line and adjacent storage zones, coinciding with rising ambient temperatures and likely increased resource availability. In April 2023 (Figure 4e), captures were again low throughout the facility. However, by June 2023 (Figure 4f), infestation levels rose markedly, particularly along the processing line and in nearby storage areas.

## 4. Discussion

The present study provides a comprehensive overview of the insect populations associated with an animal feed industry in northern Greece, documenting the diversity, abundance, and temporal dynamics of species captured over an extended monitoring period. A total of 9047 insects encompassing several major families of Coleoptera, Lepidoptera, and other insect orders known to infest stored agricultural commodities. The results highlight the dominance of a limited number of cosmopolitan stored-product pests, while a wide range of incidental species occurred sporadically. Such insect population patterns are consistent with previous research in similar industrial and storage environments, confirming that relatively few species are responsible for the majority of infestations in facilities handling plant-based materials [15,16].

The most abundant species recorded in this study were Lepidoptera adults, *T. castaneum*, *L. serricorne*, *O. surinamensis*, *S. granarius*, and *T. confusum*. These taxa collectively represented more than 85% of all insects captured. The predominance of these cosmopolitan pests aligns closely with previous findings from stored-product ecosystems throughout the Mediterranean basin and Central Europe [14,17]. The relatively high abundance of Lepidoptera adults belonging to *P. interpunctella* or *Ephestia* spp., the two most common pyralid moths associated with feed manufacturing and food processing environments, underscores the importance of flying insect species that readily disperse through production and storage areas. In addition to their prevalence in storage environments, pyralid moths such as *P. interpunctella* and *Ephestia* spp. are also well adapted to milling and feed-processing facilities, where larval webbing can accumulate on equipment and, in severe cases, clog machinery and interfere with production processes. Since the pheromone lure used in the delta traps attracts both taxa, and many insects were collected in damaged condition, species-level separation was not feasible. This limitation is common in long-term industrial monitoring programs in which sympatric pyralid species respond to the same pheromone components [16]. It is also possible that interspecific interactions between *P. intepunctella* and *Ephestia* spp., including competition for food resources or oviposition sites, may contribute to the population fluctuations observed in the present study. Similar dominance of Pyralid moths has been reported in flour mills, feed industries, and warehouses across southern Europe [16,24]. Future research incorporating species-specific pheromone lures or molecular identification methods would allow a more detailed assessment of species proportions and their respective population dynamics.

*Tribolium castaneum* and *T. confusum* are particularly well adapted to industrial environments, capable of exploiting residues in machinery, cracks, and dust accumulations, where they can persist even under suboptimal conditions [18]. Their presence in relatively high numbers within the feed facility suggests that there are continuous feeding sources and highlights their ability to colonize processed materials with reduced moisture content, which in feed production facilities typically remain below 12% to prevent microbial growth and maintain product quality.

The strong representation of *L. serricorne* and *O. surinamensis* also confirms previous studies reporting their frequent occurrence in storage and processing areas [15,16]. *Lasioderma serricorne*, although traditionally associated with tobacco, is known to infest a wide range of dried materials, including feed components, spices, and grains. *Oryzaephilus surinamensis* typically occurs in dusty, nutrient-rich microhabitats, often near machinery or packaging zones, and can proliferate rapidly under warm conditions. The consistent detection of *S. granarius* further indicates that whole or cracked grain components are present within the facility, serving as suitable feeding substrates.

In contrast, other insect species, such as *R. dominica*, *S. paniceum*, *S. oryzae*, and *Cryptolestes* sp. (Coleoptera: Laemophloeidae), were captured in lower numbers and classified as influent or recedent. These findings reflect the typical uneven distribution of insect communities in industrial settings, where the microclimatic and structural heterogeneity determines species persistence [2]. The sporadic appearance of outdoor or occasional taxa (e.g., Diptera, Formicidae, Carabidae, Hymenoptera) may be attributed to transient intrusion rather than established infestations. It is important to acknowledge that trap-based monitoring may underrepresent species with limited flight capacity or cryptic behavior. Although pheromone- and food-baited traps are effective tools for detecting highly mobile species and assessing overall population trends, weak fliers or insects that remain concealed within residues, machinery, or structural crevices may not be efficiently sampled. As a result, the composition and abundance of certain taxa may be underestimated. Future monitoring efforts in similar industrial environments would benefit from integrating complementary approaches, such as direct inspection of food residues or targeted sampling of high-risk microhabitats, to obtain a more comprehensive characterization of the facility’s insect community.

The two-year monitoring period revealed pronounced temporal variability in insect captures. Total captures peaked in early September 2021, followed by a progressive decline during autumn and winter and a secondary increase in late spring 2023. This seasonal pattern is characteristic of temperate regions, where population growth of stored-product insects is closely linked to ambient temperature and humidity [13]. In northern Greece, summer conditions often favor accelerated development and increased flight activity, while low winter temperatures limit reproduction and dispersal.

It should be noted that the frequency of pesticide applications within the facility was directly related to the observed seasonal variation in insect activity. During the warmer months, when insect captures increased markedly, treatments with Actellic 50 EC (48% AI) were applied more frequently as part of the facility’s routine pest management program. Conversely, during winter, applications were only occasional due to the markedly lower insect activity. The temporary reductions in total captures observed during certain summer periods may therefore reflect the immediate effects of these treatments on insect populations. However, the subsequent recovery of captures indicates that these effects were short-lived, likely due to the limited residual persistence of the formulation and the high reinfestation potential under industrial conditions. This pattern highlights the dynamic interplay between environmental conditions, pest pressure, and management intensity, emphasizing the importance of continuous monitoring and preventive measures to maintain effective control within feed manufacturing facilities.

Among individual species, Lepidoptera adults displayed the most pronounced seasonal peaks, with maximum captures in late summer and early autumn, consistent with reports by Agrafioti et al. [16], who found similar periodicity in Pyralid moth populations in feed industries. *Tribolium castaneum* and *L. serricorne* also showed recurrent peaks in summer and autumn, reflecting their thermophilic nature and ability to reproduce efficiently under warm conditions. *Oryzaephilus surinamensis* and *S. granarius* exhibited irregular fluctuations, suggesting that their population dynamics are influenced not only by external temperature but also by internal microhabitat conditions, such as food residues and localized humidity. *Tribolium confusum*, by contrast, was less abundant and lacked distinct seasonal trends, a pattern also observed in other long-term trapping studies [15].

The correlation analysis among dominant species revealed several positive relationships, notably between *T. castaneum* and *O. surinamensis*, and between *T. castaneum* and *S. granarius*. These associations likely reflect shared environmental preferences or co-occurrence in similar microhabitats, such as dusty accumulations of grain residues. Similar correlations between *T. castaneum* and *O. surinamensis* have been reported in other storage facilities, suggesting possible coexistence rather than competition [25]. Positive associations between *S. granarius* and Lepidoptera indicate overlapping resource use, as both groups are linked to stored grain and derived products. Conversely, weak or negative correlations among other pairs, such as *T. confusum* with *O. surinamensis*, may reflect differences in microhabitat preference or competitive exclusion under specific conditions. Although overlapping resource use can promote competition, the abundant availability of feed materials and residues within the facility suggests that direct competition for food was unlikely to be the main factor influencing the associations among species. Rather, the positive correlations observed among certain taxa may be better explained by shared microhabitat preferences, similar responses to prevailing environmental conditions, or parallel dispersal behavior within the industrial environment. These mechanisms are consistent with the complex and heterogeneous nature of feed manufacturing facilities, where multiple species can coexist without experiencing strong resource limitation. Spatial heterogeneity within the facility—arising from differences in temperature, humidity, and product type—likely contributed to the observed variation in species distribution. Previous studies demonstrated that storage structures often exhibit “hotspots” of infestation that remain stable over time, emphasizing the importance of spatially targeted monitoring [15]. Although the present study focused primarily on temporal rather than spatial analysis, the observed patterns suggest that environmental gradients within the industrial setting influenced species occurrence.

The insect populations observed in the present survey closely mirror those reported by Agrafioti et al. [16] in a similar feed industry in central Greece, where *T. castaneum*, *O. surinamensis*, and Lepidoptera dominated the captures. Likewise, Athanassiou and Buchelos [15] documented comparable dominance structures in wheat silos, with Tenebrionidae and Silvanidae as the prevailing families. However, the relative abundance of Lepidoptera adults in the current study was considerably higher than in silo environments, highlighting potential differences in product type and storage structure. Feed mills typically contain mixtures of raw and processed materials that emit volatile compounds attractive to adult moths, facilitating rapid colonization.

Seasonal fluctuations similar to those recorded here have also been observed in long-term monitoring programs in flour mills and feed facilities across southern Europe [16,26]. In these studies, the abundance of stored-product beetles increased markedly during warmer months, paralleling ambient temperature rises. The persistence of *T. castaneum* and *O. surinamensis* throughout the year indicates their ability to survive under a broad range of conditions, particularly within protected indoor environments where temperature fluctuations are moderated.

The dominance of a few key species underscores the importance of continuous surveillance and targeted management in feed industries. Monitoring data such as that obtained in the present study can provide an early warning of potential infestations, allowing the implementation of control measures before extensive contamination occurs. The pronounced seasonality of insect captures suggests that preventive actions should be intensified during late spring and summer, when populations typically increase.

Moreover, the coexistence of multiple species within the same facility highlights the necessity of integrated pest management (IPM) approaches that combine sanitation, physical exclusion, and selective application of insecticides. Monitoring programs, when properly maintained, not only inform management decisions but also serve as long-term indicators of hygiene and pest risk within the facility [11]. The results of this study further demonstrate the utility of pheromone and food-baited traps for capturing a broad spectrum of species, including both primary and secondary pests.

In summary, this study expands the knowledge of the entomological populations associated with animal feed industries in Greece, documenting the dominance of cosmopolitan stored-product insects and highlighting their temporal variability under real industrial conditions. The findings confirm that the insect species of feed mills is largely similar to that of grain storage facilities but may exhibit distinct quantitative patterns due to differences in product composition and environmental management. Future research should integrate spatial mapping of insect infestation, microclimatic monitoring, and modeling approaches to better predict insect dynamics within complex industrial environments. Such efforts will enhance the efficiency of monitoring programs and contribute to the design of more sustainable and precise pest management strategies.

## Figures and Tables

**Table 1:** Dominance and Frequency of the captured insects in the feed industry.

Family/Taxa	Species	% of the Total Number of Adults	Dominance	% of the Total Number of Samples	Frequency
Tenebrionidae	*Tribolium confusum*	5.32	Dominant	9.13	Accidental
	*Tribolium castaneum*	12.91	Dominant	15.12	Accidental
	*Latheticus oryzae*	0.51	Recedent	1.75	Accidental
	*Palorus* sp.	0.06	Recedent	0.26	Accidental
	*Alphitobius diaperinus* (adult)	0.01	Recedent	0.05	Accidental
	*Alphitobius diaperinus* (larvae)	0.01	Recedent	0.05	Accidental
Silvanidae	*Oryzaephilus surinamensis*	6.33	Dominant	11.04	Accidental
	*Ahasverus advena*	0.04	Recedent	0.21	Accidental
Anobiidae	*Lasioderma serricorne*	10.95	Dominant	6.60	Accidental
	*Stegobium paniceum*	2.11	Influent	1.24	Accidental
Bostrychidae	*Rhyzopertha dominica*	0.80	Recedent	1.75	Accidental
Curculionidae	*Sitophilus oryzae*	2.86	Influent	3.35	Accidental
	*Sitophilus granarius*	7.42	Dominant	9.34	Accidental
Laemophloeidae	*Cryptolestes* sp.	0.86	Recedent	2.01	Accidental
Cryptophagidae	*Cryptophagus* sp.	0.01	Recedent	0.05	Accidental
Cleridae	*Necrobia rufipes*	0.04	Recedent	0.31	Accidental
Dermestidae	*Attagenus* sp.	0.08	Recedent	0.31	Accidental
	Adult	0.23	Recedent	0.88	Accidental
	Larvae	0.45	Recedent	1.19	Accidental
Nititulidae	*Carpophilus* sp.	0.17	Recedent	0.77	Accidental
Ptinidae	*Ptinus* sp.	0.41	Recedent	1.14	Accidental
Lepidoptera	Adults	45.11	Dominant	19.09	Accidental
	Larvae	0.13	Recedent	0.36	Accidental
Carabidae		0.01	Recedent	0.05	Accidental
Diptera		0.24	Recedent	1.81	Accidental
Formicidae		0.04	Recedent	0.41	Accidental
Hemiptera		0.14	Recedent	0.57	Accidental
Hymenoptera		4.68	Influent	0.21	Accidental
Staphilinidae		0.06	Recedent	0.52	Accidental

**Table 2:** Correlation coefficient values for pairs of dominant insect species captures, in all cases, *df* = 50.

Pair of Dominant Insect Species	R	*T*	*P*
*T. confusum*—*T. castaneum*	0.10	−2.649	<0.01
*T. confusum*—*O. surinamensis*	−0.00	−0.668	0.50
*T. confusum—S. granarius*	0.04	−0.972	0.33
*T. confusum*—Lepidoptera	0.00	−5.381	<0.01
*T. confusum—L. serricorne*	0.06	−1.283	0.20
*T. castaneum—O. surinamensis*	0.26	2.482	0.01
*T. castaneum—S. granarius*	0.65	2.640	0.01
*T. castaneum*—Lepidoptera	0.43	−4.873	<0.01
*T. castaneum—L. serricorne*	0.33	0.462	0.64
*O. surinamensis—S. granarius*	0.48	0.660	0.51
*O. surinamensis*—Lepidoptera	0.08	−5.200	<0.01
*O. surinamensis—L. serricorne*	0.01	−1.049	0.29
*S. granarius*—Lepidoptera	0.33	−5.454	<0.01
*S. granarius—L. serricorne*	0.27	−0.843	0.40
Lepidoptera—*L. serricorne*	0.35	4.838	<0.01

## Data Availability

The original contributions presented in this study are included in the article. Further inquiries can be directed to the corresponding author.
